# Aircraft Control Parameter Estimation Using Self-Adaptive Teaching-Learning-Based Optimization with an Acceptance Probability

**DOI:** 10.1155/2021/4740995

**Published:** 2021-12-20

**Authors:** Yodsadej Kanokmedhakul, Natee Panagant, Sujin Bureerat, Nantiwat Pholdee, Ali R. Yildiz

**Affiliations:** ^1^Sustainable Infrastructure Research and Development Center, Department of Mechanical Engineering, Faculty of Engineering, Khon Kaen University, Khon Kaen, Thailand; ^2^Department of Mechanical Engineering, Bursa Uludag University, Bursa, Turkey

## Abstract

This work presents a metaheuristic (MH) termed, self-adaptive teaching-learning-based optimization, with an acceptance probability for aircraft parameter estimation. An inverse optimization problem is presented for aircraft longitudinal parameter estimation. The problem is posed to find longitudinal aerodynamic parameters by minimising errors between real flight data and those calculated from the dynamic equations. The HANSA-3 aircraft is used for numerical validation. Several established MHs along with the proposed algorithm are used to solve the proposed optimization problem, while their search performance is investigated compared to a conventional output error method (OEM). The results show that the proposed algorithm is the best performer in terms of search convergence and consistency. This work is said to be the baseline for purely applying MHs for aircraft parameter estimation.

## 1. Introduction

Flight control is one of the most important parts in developing a new aircraft or improving an existing one. It is even more crucial when the applications of unmanned aerial vehicles (UAV) have been introduced. Traditionally, aircraft motion is modelled based on the equations of motion or Newton's second law, leading to a system of nonlinear equations. Often, such a system is linearized, resulting in a linear state-space control model. In order to reach the possibly highest performance for flight control, identification of aerodynamic parameters and the aircraft dynamic model needs to be accurate. Although aircraft aerodynamic parameters including aerodynamic, stability, and control derivatives can be evaluated from an empirical model [1], numerical models (a vortex lattice method) [2], computational fluid dynamics (CFD) [3]), and wind tunnel test, errors between the test data and the real aircraft data are still inevitable. The designed aircraft and the manufactured aircraft are always different, while it is even worse when the aircraft is subject to structural flexibility in real flight. This implies that accurate system identification of the real or manufactured aircraft is always required. In this regard, parameter estimation techniques are necessary and have been one of the most popular research topics in the field of flight dynamics and control. Conventional parameter estimation techniques that can be used to evaluate stability and control derivatives from flight information have been presented [4]. However, the traditional techniques are suitable for estimating stability derivatives in a linear trim condition for a stable and rigid aircraft. They are insufficient for a highly manoeuvrable or unstable aircraft, and also expensive computation is required for estimating a large number of parameters for a full-order model of an aircraft. Also, some of the more efficient parameter estimation methods have been developed based on an implicated function technique in combination with optimization. These include the Equation Error Method (EEM) [5–8], the Output Error Method (OEM) [9–11], and the Filter Error Method (FEM) [12–15]. However, those methods still require a predefined/initial aircraft model. In this regard, development of the more efficient flight parameter estimation from flight test data without a predefined aircraft model is a challenging topic.

Recently, efficient flight parameter estimation techniques based on artificial intelligence (AI) and optimization tools have been proposed, e.g., artificial neural network (ANN) and fuzzy set theory [16–18]. The combination of AI and MH search has also been presented [19, 20]. However, even all those techniques cannot be used without a predefined aircraft model. ANN and fuzzy require a large amount of training data and also rely on efficient parameter settings. Using standalone MH for such a problem as an inverse optimization problem solver, if successful, could be a good tool for aircraft flight control.

MH (also known as evolutionary algorithms) can be classified as optimization methods which are global and nongradient optimizers. Due to such advantages, they can deal with any kind of design variable, objective, and constraint functions, although they may be less efficient in some cases. They can also explore a Pareto front within a single run, in cases of a multiobjective optimization problem. Therefore, they are at present the most used and popular optimizers for real engineering design optimization problems [21–24]. For optimization applied to an inverse problem, successful use of MHs is reported worldwide, such as a damage detection problem [25, 26], an inverse kinematic design of a robot [27], robot trajectory planning optimization [28], mechanism synthesis [29], and parameter identification of photovoltaic models [30, 31]. For inverse problem optimization of aircraft parameters estimation using MH, to our knowledge studies are rare. Some MHs found being used for solving such a problem are a classical genetic algorithm (GA) [32]. Since investigation on the use of MHs for aircraft control system identification has been limited, it is thus a motivation for this work. Up to the present time, since one of the very first algorithms GA was invented, there have been over a thousand MHs and their variants presented in the literature. A comparative performance study of some established and newly invented MHs on the parameter estimation of an aircraft system is one interesting subject while the more challenging task is to develop a new powerful MH or to improve the performance of an existing MH for such an inverse problem. Among existing MHs, teaching-learning-based optimization (TLBO) is an outstanding MH, which is found to be one of the most powerful algorithms for solving inverse problems through optimization [25, 29–31, 33]. However, its performance on the inverse problem of parameter estimation of an aircraft system has never been tested. Furthermore, although TLBO was found to be one of the best optimizers for solving inverse problems through optimization, the original version is developed for general optimization problem. Therefore, enhancing the TLBO algorithm based on a novel technique for this specific optimization problem is challenging and will lead to a powerful tool for aircraft parameter estimation.

As a result, this work proposes an efficient MH algorithm called “self-adaptive teaching-learning-based optimization with acceptance probability (SaTLBO-AP)” for solving an inverse problem of aircraft parameter estimation. The proposed algorithm is based on using TLBO as the main algorithm in combination with a diversity archive. The self-adaptive scheme exploits the acceptance probability used in simulated annealing for the balance between diversification and intensification. The inverse optimization problem for longitudinal flight parameter identification of the HANSA-3 aircraft [20] is proposed. The optimization problem is then solved by the proposed algorithm along with several newly invented and well-established MHs, including Ant Lion Optimizer (ALO) [34], Whale Optimization Algorithm (WOA) [35], Salp Swarm Algorithm (SSA) [36], Moth-Flame Optimization (MFO) [37], Grey Wolf Optimization (GWO) [38], Grasshopper Optimization Algorithm (GOA) [39], Dragonfly Algorithm (DA) [40], Water Cycle Algorithm (WCA) [41], Multi-Verse Optimizer (MVO) [42], Sine Cosine Algorithm (SCA) [43], Monarch Butterfly Optimization (MBO) [44], Slime Mould Algorithm (SMA) [45], Elephant Herding Optimization (EHO) [46], Artificial Bee Colony Algorithm (ABC) [47], Self-adaptive Differential Evolution Algorithm (SaDE) [48], Improved Teaching-Learning-Based Optimization (ITLBO) [31], and the original Teaching-Learning-Based Optimization (TLBO) [49]. The results obtained are compared and discussed.

The rest of this paper includes sections on the proposed algorithm, SaTLBO-AP, formulation of the inverse optimization problem for longitudinal flight, parameter estimation, numerical experiments, results and discussion, and conclusions.

## 2. Formulation of Inverse Optimization Problem

**Figure 1:**
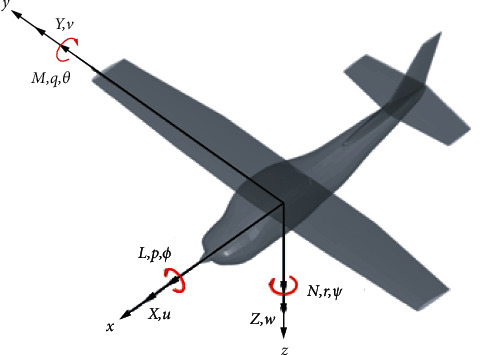
Aircraft coordinate systems.

Rigid aircraft flight dynamics are governed by the equations of motion or Newton's second law. The rigid aircraft has 6 degrees of freedom with 3 for translations and the other 3 for rotations. The conventional north-east-down coordinates can be used as an inertial reference frame. It is also convenient to use the body axes as shown in Figure 1. The equations of motion lead to a nonlinear flight dynamic model. In order to simplify the model, a small perturbation approach is employed to linearize the model. Then, with the left right symmetry of an aircraft, the model can be separated into a longitudinal and lateral/directional motions. This leads to easy to handle aircraft dynamic and control models.

In order to examine the performance of the proposed parameter estimation approach, parameter estimation of the longitudinal flight control model is presented. The nonlinear flight control model for longitudinal motion of a conventional aircraft used in this study can be expressed as [4]

(1) $$\begin{array}{c} C_{D}=C_{D_{0}+}C_{D_{\alpha }}\alpha +C_{D_{\delta e}}\delta e, \end{array}$$

(2) $$\begin{array}{c} C_{L}=C_{L_{0}}+C_{L_{\alpha }}\alpha +C_{L_{q}}\left(\frac{\bar{q}\bar{c}}{2V}\right)+C_{L_{\delta e}}\delta e, \end{array}$$

(3) $$\begin{array}{c} C_{m}=C_{m_{0}}+C_{m_{\alpha }}\alpha +C_{m_{q}}\left(\frac{\bar{q}\bar{c}}{2V}\right)+C_{m_{\delta e}}\delta e, \end{array}$$

(4) $$\begin{array}{c} \dot{V}=-\left(\frac{\bar{q}S}{m}\right)C_{D}+g\;\sin\left(\alpha -\theta \right)+\left(\frac{F_{\text{eng}}}{m}\right)\cos\left(\alpha \right), \end{array}$$

(5) $$\begin{array}{c} \dot{\alpha }=-\left(\frac{\bar{q}S}{mV}\right)C_{L}+q+\left(\frac{g}{V}\right)\cos\left(\alpha -\theta \right)-\left(\frac{F_{\text{eng}}}{mV}\right)\sin\left(\theta \right), \end{array}$$

(6) $$\begin{array}{c} \dot{\theta }=q. \end{array}$$

The aerodynamic parameters  Θ={*C*_*D*0_, *C*_*Dα*_, *C*_*D*_*δe*__, *C*_*L*_0__,  *C*_*L*_*α*__, *C*_*L*_*q*__, *C*_*L*_*δe*__, *C*_*m*_0__, *C*_*m*_*α*__, *C*_*m*_*q*_,_*C*_*m*_*δe*__} are assumed unknown and to be identified.

To apply an inverse optimization problem for longitudinal motion parameter estimation, the design problem is posed to find a set of aerodynamic parameters, in order to minimise errors between the longitudinal response of a real aircraft and the calculated response from the longitudinal dynamic equations. The optimization problem can be expressed as

(7) $$\begin{array}{c} \min:f\left(x\right)=\overset{l}{\underset{i=1}{\sum }}\overset{t_{\text{end}}}{\underset{t=t_{0}}{\sum }}\frac{\left|r_{\text{real},i}\left(t\right)-r_{\text{estimate},i}\left(t\right)\right|}{\left|r_{\text{real},i}\left(t\right)\right|}. \end{array}$$

Subject to

(8) $$\begin{array}{c} L_{b}\leq x\leq U_{b}, \end{array}$$

where *x*=Θ is a vector of design variables having *L*_b_ and *U*_b_ as the lower and upper bounds. The details of the design variables are shown in Table 1. The parameters *r*_real,*i*_ and *r*_estimate,*i*_ are respectively the *i*^th^ real and estimated longitudinal motion parameter time responses, whereas *l* is the number of longitudinal parameters considered. Four longitudinal parameters include *V*, *α*, *θ*,   and *q*. The parameters *t*_0_ and *t*_end_ are the initial and final times of the simulation, respectively.

As there are several types of physical parameters with totally different units, e.g., aircraft velocity and angular position, each estimation error in (7) is therefore normalised before being summed up. This step can be easily achieved by dividing by the absolute value of the real time response.

**Figure 2:**
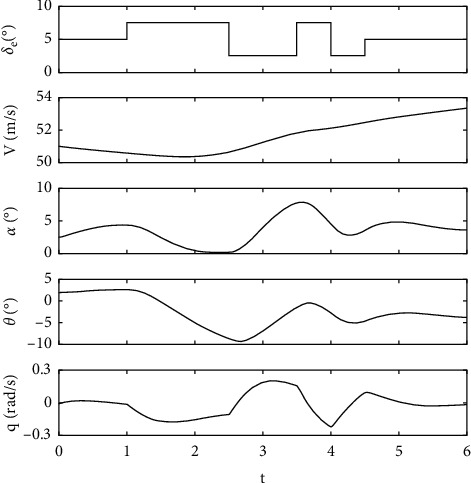
The state time response used as real flight data.

In this study, the real time response from a flight test is simulated using the flight model of the HANSA-3 aircraft as shown in Table 2, while the target values of aerodynamic parameters {*C*_*D*0_, *C*_*Dα*_, *C*_*D*_*δe*__, *C*_*L*_0__,  *C*_*L*_*α*__, *C*_*L*_*q*__, *C*_*L*_*δe*__, *C*_*m*_0__, *C*_*m*_*α*__, *C*_*m*_*q*_,_*C*_*m*_*δe*__} are shown in Table 3. The aircraft flight data are simulated with the elevator deflection of a 3-2-1–1 step input. The simulation is performed for a six-second time length (*t*) with a time step (Δ*t*) of 0.025 second. Gaussian noise with zero mean is added to the system response with the degree of noise at 0%, 5%, and 10% with respect to the amplitude of the time response. The state time response of the longitudinal motion **r**={*V*, *α*, *θ*, *q*}^*T*^ can be numerically solved based on (9), while $\dot{r}$ can be calculated based on (1)–(6). The state time response used as real flight data is shown in Figure 2.

(9) $$\begin{array}{c} r\left(t+\Delta t\right)=r\left(t\right)+\int_{t}^{t+\Delta t}\dot{r}\left(t\right)dt. \end{array}$$

## 3. Self-Adaptive Teaching-Learning Based Optimization with an Acceptance Probability

A TLBO is a simple but efficient MH proposed by Rao et al. in 2011 [49]. The algorithm was inspired by the behaviour of teaching and learning in a class. The main search procedure of the TLBO consists of population initialisation, reproduction, and selection, while, at the reproduction process, there are two main phases called the teaching and learning phases. Each individual in the population is first updated in the teaching phase with the relation.

(10) $$\begin{array}{c} x_{\text{teaching}}^{i}=x^{i}+ran\;d\left\{x_{\text{teacher}}-\left(T_{F}x_{\text{mean}}\right)\right\}, \end{array}$$

where *x*^*i*^ = the *i*th individual in the population. *x*_teacher_ = the best individual. *x*_mean_ = mean value of other members in the population. rand = *a* uniform random number in the range of [0, 1]. *T*_F_ = teaching factor, which can be either 1 or 2 at random.

The offspring and parents are then put together while the best of them is selected and sent to the learner phase. In the learner reproduction phase, a particular offspring can be created as

(11) $$\begin{array}{c} x_{learner}^{i}=\left\{\begin{array}{cc} x^{i}+rand\left(x_{i1}-x_{i2}\right), & \text{if }f\left(x_{i1}\right)<f\left(x_{i2}\right),\\ x^{i}+rand\left(x_{i2}-x_{i1}\right), & \text{if }f\left(x_{i2}\right)<f\left(x_{i1}\right), \end{array}\right.\; \end{array}$$

where *x*_*i*1_ and *x*_*i*2_ are two randomly selected individuals in the population. The greedy selection is then performed in the same manner as with the teaching phase. The computational steps are given in Algorithm 1.

From Algorithm 1, the original TLBO teaching phase is performed using one teacher (the current best solution), while the learning phase is performed exploiting two randomly selected students (individuals). This, to some extent, leads to limitation in TLBO search exploration and exploitation. Therefore, this work proposed an improved version of TLBO by introducing several numerical schemes. For the proposed self-adaptive teaching-learning based optimizer with acceptance probability algorithm, both teaching and learner phases are upgraded. Multiple teachers are assigned in the teacher phase while a three-student learning scheme is added to the learner phase to enhance its convergence rate. With several new numerical schemes being added, some control parameters are exploited in the new algorithm. While the original TLBO is said to be a derivative-free MH, the proposed algorithm applies self-adaptive strategies to the added control parameters.

In the teaching phase, a diversity archive is used to keep some promising solutions which have good balance between exploration and exploitation. Those solutions are assigned as teachers. The archive is created and updated using the nondominated sorting technique to classify solutions to the archive. The nondominated sorting is operated based on simultaneously minimising the original objective function (*f*(*x*)) and the diversity objective function (*f*_D_(*x*)) [29]. The diversity function values are calculated based on a combination of the population in the current iteration and the populations from a few previous iterations. Then, the diversity function of those individuals can be computed as

(12) $$\begin{array}{c} f_{D}\left(x_{i}\right)=w_{1}f\left(x_{i}\right)+w_{2}f_{2}\left(x_{i}\right), \end{array}$$

where *w*_1_ and *w*_2_ are weighting coefficients with the condition *w*_1_ + *w*_2_ = 1, while *w*_1_ is generated randomly within the range of [0,1]. The function *f*_2_ can be calculated based on (11)

(13) $$\begin{array}{c} f_{2}\left(x_{i}\right)=\overset{n_{P}}{\underset{j=1}{\sum }}\frac{1}{\max\left(0.0001,D_{ij}\right)}. \end{array}$$

The variable *D*_*ij*_ is a Eulerian distance between individuals *i* and *j*, while max(0.0001, *D*_*ij*_) is the maximum value between 0.0001 and *D*_*ij*_ which is used to avoid a singularity occurring in the calculation. *n*_*P*_ is the number of individuals in the pool. (11) is still used for the teaching reproduction phase, but the teacher can be selected between the best solution and those in the diversity archive with a given probability. The selection of the *x*_*teacher*_ is performed based on a probability of selection which can be expressed as

(14) $$\begin{array}{c} x_{\text{teacher}}=\left\{\begin{array}{cc} x_{\text{best}}, & \text{if rand}<p_{T},\\ x_{D\text{,rand}}, & \text{otherwise,} \end{array}\right. \end{array}$$

where *p*_T_ is the probability of selecting the best solution, while **x**_*D*,*ran*  *d*_ is an individual randomly selected in the diversity archive (archive of nondominated solutions obtained from *f*_D_ and *f*_2_). The probability of selecting the best solution for the teaching reproduction is made self-adaptive based on the accumulated data on each optimization run. Three subintervals for generating *p*_T_ are defined as [0.4, 0.5], [0.5, 0.6], and [0.6, 0.7] where selection of the intervals is carried out using a roulette wheel selection technique. For example, if subinterval one is selected, the value of *p*_T_ is generated as

(15) $$\begin{array}{c} p_{T}=0.4+\text{rand}\left(0.5-0.4\right), \end{array}$$

where rand ∈ [0,1] is a uniform random number. The *j*-th subinterval has the probability of being selected as *p*_wj_, which can be computed from

(16) $$\begin{array}{c} p_{wj}=\frac{p_{T_{\text{success}},j}}{p_{T_{\text{success}},j}+p_{T_{\text{fail}},j}}. \end{array}$$

**Figure 3:**
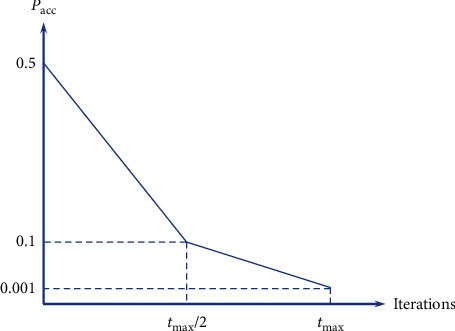
Acceptance probability scheduling.

Initially, two 1 × 3 vectors *p*_T_success_ and *p*_T_fail_ whose elements are all zeroes are created. During the teaching reproduction, if the *j*-th subinterval is used to generate the value of *p*_T_ and the reproduced offspring is better or as good as its parent, the value of *p*_T_success,j_ should be increased by adding a point to its *j*-th element. On the other hand, if it fails to surpass its parent, the value of *p*_T_fail,j_ should be increased by one point. With such a concept, the value of *p*_wj_ depends on the history of its use being either a success or a failure. Nevertheless, counting only success or failure can possibly lead to local optimum traps of the evolution of *p*_wj_; as a result, the acceptance probability concept is used similarly to the Boltzmann's probability employed in simulated annealing. As a result, in cases that the *j*-th subinterval is used leading to an offspring *x*^*i*^_teaching_, the updating scheme for both *p*_T_success,j_ and *p*_T_fail,j_ can be expressed as

(17) $$\begin{array}{c} f\left(x_{\text{teaching}}^{i}\right)\leq f\left(x^{i}\right),\\ p_{T\_\text{success},j}=p_{T\_\text{success},j}+1,\\ \text{Else,}\\ \text{If rand}<p_{\text{acc}},\\ p_{T\_\text{success}}=p_{T\_\text{success},j}+0.5,\\ \text{Else,}\\ p_{T\_\text{fail},j}=p_{T\_\text{fail},j}+1, \end{array}$$

where *p*_acc_ is an acceptance probability set with a high value initially and reduced as the optimization run progresses. Although it has failed, the *p*_T_success,j_ still has a chance to increase its score to 0.5 if the acceptance probability is passed. In this work, simple probability scheduling as displayed in Figure 3 is used, where *t*_MAX_ is the maximum iteration number.

For the learner phase, the 2-student learning in (11) is still used along with the 3-student learning strategy [33]. The selection of the two learning strategies relies on the probability of choosing the 2-student learning defined as *p*_L_. The 3-student learning is achieved by randomly selecting three individuals in the current population, then the search direction is computed in such a way that two other students are directed towards the best student. The new learner reproduction can then be written as

(18) $$\begin{array}{c} x_{\text{learner}}^{i}=\left\{\begin{array}{cc} \text{Eq}.\left(10\right), & \text{ if rand}<p_{L},\\ x^{i}+\text{rand}\left(x_{i3}-x_{i1}\right)+ran\;d\left(x_{i3}-x_{i2}\right), & \text{otherwise,} \end{array}\right. \end{array}$$

where *x*_*i1*_, *x*_*i2*,_*x*_*i3*_ are randomly selected from the current population and the last one is the best of them. The variable *p*_L_ is generated in a similar way to *p*_*T*_. That means there are three subintervals for randomly generating *p*_*L*_, while the 1 × 3 vectors *p*_L_success_ and *p*_L_fail_ are used to memorize the successful and failed records of using the *j*-th subinterval. Similarly, the probabilities for the roulette wheel selection are computed using (16).

The search process of SaTLBO-AP starts with initialising a population, a schedule of *p*_*acc*_, and the initial (zero) sets of *p*_T_success_, *p*_T_fail_*p*_L_success_ and *p*_L_fail_. After objective functions of the current population are evaluated, the diversity archive is created. Then, the reproduction process with the teaching and learner phases is performed, and the *p*_T_success_, *p*_T_fail_*p*_L_success_ and *p*_L_fail_ sets and the parameter *p*_*acc*_ are updated. The search process is repeated until the termination criterion is satisfied. The computational steps of the proposed SaTLBO-AP are shown in Algorithm 2.

## 4. Numerical Experiment

To examine the search performance of the proposed algorithm for solving aircraft parameter estimation, the proposed aircraft longitudinal parameter estimation problem in Section 2 was used. The random noise at 0%, 5%, and 10% levels is added into the real flight data, leading to the three cases of the test problem. A number of established MHs along with the proposed algorithm are used to solve such a problem. The MHs used in this study include the following:

Ant Lion Optimizer (ALO) [33], Dragonfly Algorithm (DA) [40], Grasshopper Optimization Algorithm (GOA) [39], Grey Wolf Optimization (GWO) [38], Moth-Flame Optimization Algorithm (MFO) [37], Multi-Verse Optimizer (MVO) [42], Sine Cosine Algorithm (SCA) [43], Salp Swarm Algorithm (SSA) [36], Water Cycle Algorithm (WCA) [41], Whale Optimization Algorithm (WOA) [35], Monarch Butterfly Optimization (MBO) [44], Slime Mould Algorithm (SMA) [45], Elephant Herding Optimization (EHO) [46], Artificial Bee Colony Algorithm (ABC) [47], Self-adaptive Differential Evolution Algorithm (SaDE) [48], Teaching-Learning Based Optimization (TLBO) [49], Improved Teaching-Learning-Based Optimization (ITLBO) [31], and the proposed algorithm (SaTLBO-AP).

Each optimizer is used to solve the problems for 20 independent runs. The population size and maximum number of iterations are set to be 200 and 250, respectively. For any optimizer using a different population size, they will be terminated at the same number of function evaluations (FEs) of 200 × 250 = 50,000 FEs.

In addition, the conventional OEM has been used to compare with the MH approach. The OEM used in this study is based on the default code from the System Identification Program for Aircraft (SIDPAC) from NASA [50]. Due to the requirement of a predefined initial solution of OEM which is difficult to determine in the real situation, 20,000 initial solutions are generated based on the Latin hypercube sampling in this study. As a result, 20,000 runs from 20,000 initial solutions are performed for the OEM and the results obtained are discussed and compared with the proposed MH. In cases that the solution cannot converge, the OEM search process is terminated at 3,000,000 FEs. It should be noted that the numerical experiment is conducted via MATLAB 2020a with AMD Ryzen 9 5950 × 16-Core Processor 3.40 GHz with 32 GB of ram.

## 5. Results and Discussion

Having performed 20 independent runs of all MHs for solving the three cases of the optimization for aircraft control parameter estimation, the results are reported in Table 4. Table 4 shows the root mean square error (RMSE) between the longitudinal response of the real aircraft and the calculated response from the longitudinal dynamic equations at the optimum points. The mean values of the RMSE (Mean) are used to measure the MHs search convergence while the standard deviation values (Std) are used to measure MH search consistency. The results show that, for each case of the design problem, the proposed SaTLBO-AP algorithm is the best performer based on the Friedman ranking, search convergence, and search consistency, while the second best and third best algorithms are SaDE and WCA, respectively.

**Figure 4:**
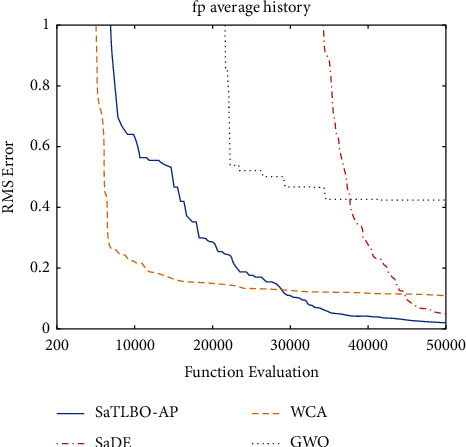
Average fitness RMSE values of 20 individual runs without noise from the top 4 algorithms.

Figure 4 shows the search history of the top four algorithms as plots of iterations versus average objective function values. It was found that WCA shows the fastest convergence while SaDE is the slowest from the beginning. Afterwards, WCA gets trapped at a local optimum after about 10,000 FEs. The proposed SaTLBO-AP seems to converge slower than the WCA initially, however, after 30,000 FEs, the proposed SaTLBO-AP is superior to the WCA and steadily approaches the global optimum. At the end of the optimization run, SaDE managed to reach near the global minimum point, but is still behind SaTLBO-AP. It can be concluded that the proposed SaTLBO-AP is obviously the best algorithm for this problem, is the most robust, and has good balance between search intensification and diversification.

Table 5 shows the top 10 best solutions obtained from 20,000 initial solution of OEM. From the table, it is seen that for the parameter estimation problem without noise, there are only 7 of 20,000 initial solutions where the RMSEs are lower than 1. There are only 4 out of 20,000 and 9 of 20,000 initial solutions with RMSE lower than 1 for the cases of 5% and 10% noise, respectively. When comparing the best obtained solution based on RMSE of the OEM and the proposed SaTLBO-AP, the OEM seems to have a slightly better RMSE for all cases, due to the use of a gradient-based optimizer. Nevertheless, such solutions can be found only 4–9 times out of 20,000 trials. When comparing the average RMSE obtained from 20 independent runs of the proposed SaTLBO-AP and average RMSE obtained from the top 10 best results classified from 20,000 solutions by using OEM, it is clearly seen that the proposed SaTLBO-AP is better for all cases. Although OEM is advantageous in terms of convergence rate with the use of gradient information, it requires a good predefined initial solution in order to obtain an acceptable solution, which is difficult to identify in a real situation. Based on this study, the possibility to obtain good results is lower than 10/20,000. However, for the proposed SaTLBO-AP based on solving the proposed inverse optimization problem of aircraft parameter estimation, an acceptable solution can be obtained for all trials without the barrier of predefining an initial solution.

**Figure 5:**
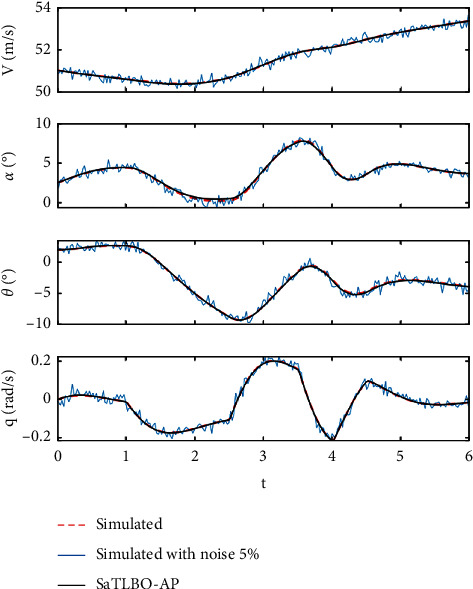
Comparison between simulated data with noise 5% and without noise from SaTLBO-AP best result.

**Figure 6:**
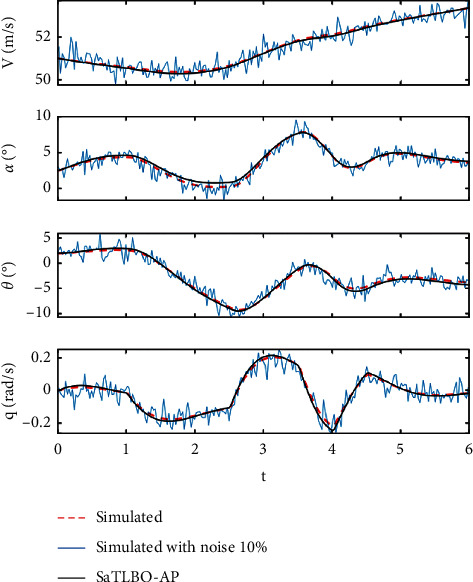
Comparison between simulated data with noise 10% and without noise from SaTLBO-AP best result.

Table 6 shows the comparison between the aerodynamic coefficients and derivatives obtained from using the three best MHs, i.e., SaTLBO-AP, SaDE and WCA and the target values. The best obtained values (best run) and standard deviation (in parentheses) are presented in the table for each aerodynamic parameter. The results reveal that the aerodynamic coefficients and derivatives obtained from the proposed SaTLBO-AP and SaDE are close to the target values for all cases. Most of the standard deviation values for all aerodynamic coefficients and derivatives obtained from SaTLBO-AP are the lowest for all cases. The output responses obtained from the best run of the proposed SaTLBO-AP compared with target output responses are plotted in Figures 5 and 6.

Overall, it was found that the proposed SaTLBO-AP is the best performer for the case studies of aircraft parameter estimation. Applying the diversity technique, parameter self-adaption and the acceptance probability concept can increase the MH search performance. The obtained values of the aerodynamic coefficients and derivatives may be slightly different from the actual or desired values; however, if a robust controller is used, such uncertainties can be coped with.

## 6. Conclusions

In this work, a new self-adaptive TLBO is proposed for aircraft parameter estimation. The method is based on integrating a diversity archive and a 3-student learning scheme into the teaching and learner phases respectively. A new self-adaptive strategy with the use of an acceptance probability is proposed. An inverse optimization problem is presented for aircraft longitudinal parameters estimation. The problem is posed to find longitudinal aerodynamic parameters by minimising errors between real flight data and those calculated from the dynamic equations. Several established MHs along with the proposed algorithm are used to solve the proposed optimization problem. The results shown that the top three best algorithms are SaTLOB-AP, SaDE, and WCA, while the proposed SaTLBO-AP is the best performer in both search convergence and consistency. When comparing the proposed SaTLBO-AP with the conventional OEM, the OEM gives better results if a good initial solution is used. Nevertheless, based on this study, only 1 of 20,000 trials for initial solutions of OEM leads to slightly better results than the proposed SaTLBO-AP, while the proposed SaTLBO-AP can obtain acceptable solutions without worrying about an initial guess. However, the proposed algorithm is shown to be suitable for the inverse problem of aircraft parameter estimation, while its performance on other inverse problems still needs investigation. This work is proposed to be the baseline of an investigation that only applies MHs for aircraft parameter estimation. For future work, performance enhancement of the proposed MH for solving the problem and for full model aircraft system identification should be studied.

## Figures and Tables

**Algorithm 1:**
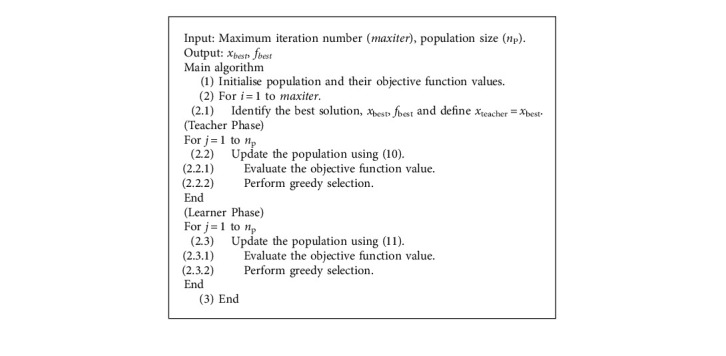
TLBO.

**Algorithm 2:**
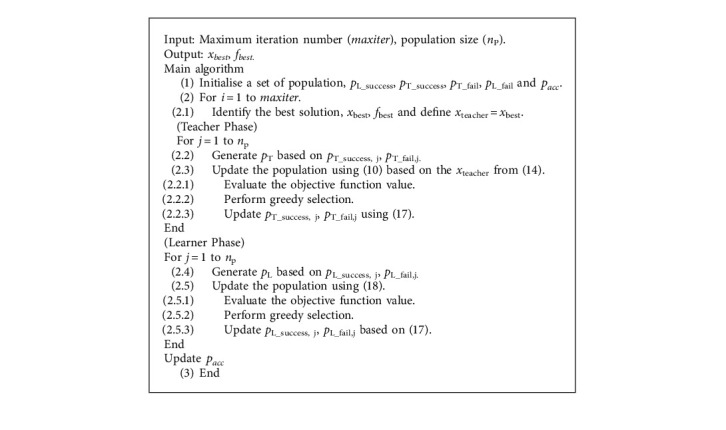
Algorithm 2 SaTLBO-AP.

**Table 1:** Design variables with lower and upper bounds.

	*C*_*D*_0__	*C*_*D*_*α*__	*C*_*D*_*δe*__	*C*_*L*_0__	*C*_*L*_*α*__	*C*_*L*_*q*__	*C*_*L*_*δe*__	*C*_*m*_0__	*C*_*m*_*α*__	*C*_*m*_*q*__	*C*_*m*_*δe*__
*L*_*b*_	0	0	0	0	0	0	0	0	−5	−50	−5
*U*_*b*_	5	1	5	5	50	200	5	1	0	0	0

**Table 2:** Flight condition and aircraft geometry of HANSA-3.

Variable	Value
Mean aerodynamic chord ($\bar{c}$)	1.21 *m*
Wingspan (*b*)	10.84 *m*
Reference wing area (*S*)	12.47 *m*^2^
Mass (*m*)	758 *kg*
True air speed (*V*)	52 *m*/*s*
Moment of inertia (*I*_*y*_)	925 *kg* *m*^2^
Engine thrust (*F*_eng_)	1136 *N*

**Table 3:** The target value of aerodynamic parameters.

Parameter	*C*_*D*_0__	*C*_*D*_*α*__	*C*_*D*_*δe*__	*C*_*L*_0__	*C*_*L*_*α*__	*C*_*L*_*q*__	*C*_*L*_*δe*__	*C*_*m*_0__	*C*_*m*_*α*__	*C*_*m*_*q*__	*C*_*m*_*δe*__
True value	0.036	0.061	0.152	0.23	4.886	37.259	0.376	0.091	–0.412	–8.792	–0.735

**Table 4:** Best, worst, average, standard deviation, and Friedman test of the RMSE at 50,000 function evaluations of 20 individual runs, with and without added noise

Algorithms	Without noise					Noise 5%					Noise 10%				
	Worst	Best	Mean	Std	FR	Worst	Best	Mean	Std	FR	Worst	Best	Mean	Std	FR
ALO	9.0037	0.1529	2.7785	3.3000	8.95	10.2319	0.2295	1.7926	2.7769	7.8	9.0889	0.4015	1.1421	2.1683	5.15
DA	25.1420	0.2640	10.0351	6.6882	14.45	28.0098	0.3375	11.9031	8.3283	14.45	16.8180	0.4513	5.9119	4.8094	13.05
GOA	28.0936	0.9277	18.0601	6.7818	16.85	28.9044	0.8945	20.7342	7.0532	17.2	26.8451	9.1712	20.9930	5.5621	17.35
GWO	1.2739	0.1994	0.3928	0.3098	5.6	1.0688	0.2304	0.4032	0.2196	5.2	0.8363	0.4266	0.5077	0.1188	5.3
MFO	9.2415	0.1832	2.6290	2.7619	9.55	4.6958	0.2581	1.6013	1.5389	8.8	9.4441	0.4307	1.5822	2.1702	8.4
MVO	29.4382	5.4741	17.8161	6.6723	16.6	25.0409	1.6569	14.0516	6.9486	16.3	29.7928	9.0117	16.8057	5.0023	16.8
SCA	8.8118	0.2209	1.4037	1.9144	8.9	3.1936	0.3810	1.4519	0.8058	10.4	2.9651	0.5678	1.3645	0.7551	10.7
SSA	0.8091	0.3462	0.6309	0.1288	7.75	0.7704	0.5509	0.6597	0.0601	7.75	0.9014	0.5689	0.7121	0.0786	8.4
WCA	0.3967	0.0119	0.1100	0.0960	2.9	0.3726	0.1909	0.2217	0.0470	2.65	0.5814	0.3943	0.4338	0.0523	3.1
WOA	24.2830	0.2409	7.9811	8.2753	11.95	24.2636	0.3772	8.9912	7.3915	14.15	20.2952	0.5557	7.1401	6.0350	13.8
MBO	22.7703	0.4050	3.3462	5.5273	10.85	15.0105	0.6030	2.0042	3.1841	10.5	26.3406	0.5751	6.9586	8.7916	13
SMA	11.2174	0.3254	2.1315	2.3528	10.8	13.6034	0.4805	2.4623	3.1702	11.3	25.2341	0.7177	3.3992	6.2542	11.1
EHO	4.0977	0.7582	1.8700	0.7765	11.25	3.4694	0.4009	1.3911	0.7981	10.55	1.1310	0.9659	1.0707	0.0423	10.85
ABC	16.4939	2.3997	7.5099	4.2127	14.6	15.2516	0.9968	5.5834	4.6919	13.5	8.6376	1.0979	3.9645	2.6187	13.35
SaDE	0.1521	0.0028	0.0488	0.0362	2.25	0.2123	0.1919	0.1975	0.0060	2.4	0.4879	0.3977	0.4253	0.0222	2.95
TLBO	4.3630	0.1490	1.7028	1.2234	10.15	6.2615	0.3263	1.6474	1.4422	10.4	3.0237	0.4984	1.3931	0.7821	10.5
ITLBO	1.5246	0.0174	0.4906	0.3604	6.35	2.5530	0.2082	0.5442	0.5402	6.45	1.1887	0.4174	0.5755	0.1820	6.05
SaTLBO-AP	**0.0369**	**0.0024**	**0.0195**	**0.0083**	**1.25**	**0.1968**	**0.1899**	**0.1914**	**0.0017**	**1.2**	**0.4043**	**0.3960**	**0.3994**	**0.0016**	**1.15**

^*∗*^FR is the Friedman test score; lower is better.

**Table 5:** Top 10 best RMSE obtained from 20,000 initial solutions of data with and without noise obtained from OEM.

No.	Without noise	Noise 5%	Noise 10%
1	3.32E-15	0.1896	0.3911
2	1.16*E* − 14	0.4020	0.3911
3	0.4325	0.5277	0.3911
4	0.6074	0.8364	0.3911
5	0.7240	2.4400	0.3911
6	0.9079	2.5514	0.4594
7	0.9877	3.5373	0.4717
8	1.9814	3.5979	0.5643
9	2.1582	4.0832	0.6481
10	3.0534	5.1517	1.9992
Best RMSE obtained from OEM	**3.32 ** *E* − **15**	**0.1896**	**0.3911**
Best RMSE obtained from SaTLBO-AP	0.0024	0.1899	0.3960
Average RMSE of the top 10 best obtained from OEM	1.0853	2.3317	0.6098
Average RMSE from SaTLBO-AP	**0.0195**	**0.1914**	**0.3994**

**Table 6:** Hansa-3 aerodynamics coefficient simulated without noise, noise 5% and 10%.

True value		Without noise			Noise 5%			Noise 10%		
		SaTLBO-AP	SaDE	WCA	SaTLBO-AP	SaDE	WCA	SaTLBO-AP	SaDE	WCA
*C*_*D*_0__	0.036	0.0367	0.0368	0.0437	0.038	0.0463	0.0544	0.0403	0.023	0
		(0.0079)	(0.0086)	(0.0253)	(0.009)	(0.0058)	(0.0227)	(0.0089)	(0.0076)	(0.0172)
*C*_*D*_*α*__	0.061	0.0587	0.0511	0.0183	0.0703	0.0433	0.0002	0.0368	0.1879	0.8955
		(0.0558)	(0.054)	(0.2957)	(0.0544)	(0.0456)	(0.273)	(0.0566)	(0.1109)	(0.3237)
*C*_*D*_*δe*__	0.152	0.1454	0.1486	0.0972	0.1366	0.065	0	0.1611	0.2274	0
		(0.0532)	(0.0721)	(0.2322)	(0.0638)	(0.0511)	(0.2145)	(0.0698)	(0.0868)	(0.2046)
*C*_*L*_0__	0.23	0.2297	0.2302	0.2384	0.2333	0.2193	0.126	0.2416	0.2005	0.0001
		(0.0037)	(0.0077)	(0.1389)	(0.015)	(0.0173)	(0.1284)	(0.0178)	(0.0504)	(0.1133)
*C*_*L*_*α*__	4.886	4.9015	4.8831	4.938	5.053	5.1848	5.3335	4.846	4.1228	5.5128
		(0.0295)	(0.1079)	(0.814)	(0.066)	(0.0597)	(0.7129)	(0.1371)	(0.5722)	(1.0446)
*C*_*L*_*q*__	37.259	37.1573	37.281	34.3262	39.5509	41.9119	48.286	43.377	57.2311	87.3309
		(1.2757)	(2.1063)	(56.1791)	(2.9128)	(2.2761)	(37.4155)	(6.5266)	(12.6068)	(50.7457)
*C*_*L*_*δe*__	0.376	0.3755	0.376	0.2488	0.1776	0.2639	1.2234	0.0033	1.0604	2.1766
		(0.0406)	(0.0672)	(1.2566)	(0.1403)	(0.1831)	(1.0458)	(0.1661)	(0.3744)	(0.9867)
*C*_*m*_0__	0.091	0.091	0.091	0.0906	0.0922	0.0944	0.0918	0.1035	0.0969	0.1037
		(0.0002)	(0.0008)	(0.0121)	(0.0005)	(0.001)	(0.0108)	(0.0009)	(0.0054)	(0.0392)
*C*_*m*_*α*__	−0.412	−0.4132	−0.4119	−0.4151	−0.4329	−0.4186	−0.4239	−0.4652	−0.3811	−0.517
		(0.0014)	(0.0037)	(0.1517)	(0.004)	(0.0082)	(0.0933)	(0.0118)	(0.0456)	(0.2)
*C*_*m*_*q*__	−8.792	−8.758	−8.8043	−8.6207	−8.6012	−8.8541	−8.1431	−10.2257	−10.221	−7.5769
		(0.0676)	(0.2355)	(3.3145)	(0.1825)	(0.3182)	(2.143)	(0.4649)	(1.3053)	(10.9678)
*C*_*m*_*δe*__	−0.735	−0.7344	−0.7355	−0.7288	−0.73	−0.763	−0.7343	−0.8156	−0.8112	−0.7767
		(0.0019)	(0.0093)	(0.0954)	(0.0063)	(0.0093)	(0.073)	(0.0153)	(0.055)	(0.37)

## Data Availability

The data used to support the findings of this study are available from the corresponding author upon request.

## Nomenclature

*C*_*D*_, *C*_*L*_, *C*_*m*_: Drag, lift, pitching moment coefficients
*C*_*D*_0__, *C*_*L*_0__, *C*_*m*_0__: Trim drag, lift, pitching moment coefficients
*p*, *q*, *r*: Angular velocities in roll, pitch, and yaw axes
*ϕ*, *θ*, *ψ*: Roll, pitch, and yaw angles
*u*, *v*, *w*: Airspeed components in x, *y,* and *z* axes
*I*_*x*_, *I*_*y*_, *I*_*z*_: Moment of inertia about x, *y*, and *z* axes
Θ: Vector of unknown parameters
*α*: Angle of attack
*C*_*Dα*_, *C*_*D*_*δe*__: ∂*C*_*D*_/∂*α*∂*C*_*D*_/∂*δ*_*e*_
*L*, *M*, *N*: Rolling, pitching, and yawing moments
$\bar{q}$: Dynamic pressure
*ρ*: Air density
*V*: True airspeed
*F*_eng_: Engine thrust.
